# Impact of Tumor Cell Cytoskeleton Organization on Invasiveness and Migration: A Microchannel-Based Approach

**DOI:** 10.1371/journal.pone.0008726

**Published:** 2010-01-15

**Authors:** Claudio G. Rolli, Thomas Seufferlein, Ralf Kemkemer, Joachim P. Spatz

**Affiliations:** 1 Department New Materials and Biosystems, Max Planck Institute for Metals Research, Stuttgart, Germany; 2 Department Biophysical Chemistry, University of Heidelberg, Institute for Physical Chemistry, Heidelberg, Germany; 3 Department für Innere Medizin, Universitätsklinik und Poliklinik für Innere Medizin I, Halle (Saale), Germany; 4 Max Planck Institute for Metals Research, ZWE Biomaterialien, Stuttgart, Germany; The Beatson Institute for Cancer Research, United Kingdom

## Abstract

Cell migration is a fundamental feature of the interaction of cells with their surrounding. The cell's stiffness and ability to deform itself are two major characteristics that rule migration behavior especially in three-dimensional tissue. We simulate this situation making use of a micro-fabricated migration chip to test the active invasive behavior of pancreatic cancer cells (Panc-1) into narrow channels. At a channel width of 7 µm cell migration through the channels was significantly impeded due to size exclusion. A striking increase in cell invasiveness was observed once the cells were treated with the bioactive lipid sphingosylphosphorylcholine (SPC) that leads to a reorganization of the cell's keratin network, an enhancement of the cell's deformability, and also an increase in the cell's migration speed on flat surfaces. The migration speed of the highly deformed cells inside the channels was three times higher than of cells on flat substrates but was not affected upon SPC treatment. Cells inside the channels migrated predominantly by smooth sliding while maintaining constant cell length. In contrast, cells on adhesion mediating narrow lines moved in a stepwise way, characterized by fluctuations in cell length. Taken together, with our migration chip we demonstrate that the dimensionality of the environment strongly affects the migration phenotype and we suggest that the spatial cytoskeletal keratin organization correlates with the tumor cell's invasive potential.

## Introduction

Cell migration is an essential characteristic of both physiological and pathological processes within the human body. Most of the current knowledge about cell migration was obtained by model studies on flat two-dimensional (2D) surfaces. However, recent studies on cell migration mechanisms revealed drastic differences in migration behaviors depending on the dimensionality of the cell environment [1]. Migration characteristics of cells, such as the speed, were found to be very different in 2D and 3D environments, and even the underlying mechanisms can be changed. Lämmermann *et al*., for example, reported that cells switch to an adhesion-independent mechanism when seeded in a three-dimensional (3D) in contrast to an adhesion-dependent migration on a flat surface [2]. In particular, migration of tumor cells has been extensively studied due to its importance in the process of cancer metastasis [3], [4]. Tumor cells in a primary tumor are able to abandon their initial environment and migrate through the surrounding parenchyma, enter the circulatory system and invade other, healthy tissues. On this journey, the cells need to regulate their migratory and invasive behavior and are exposed to a variety of mechanical interactions like shear stress and deformation [5], [6]. Remarkably, it was reported that upon pharmacological inhibition of matrix metalloproteinases, cells, instead of proteolytically degrading their local environment, were forced to “squeeze” through a collagen fiber network accompanied by drastic cell and nuclear deformation upon migration [7]. These deformations require substantial reorganization of the cytoskeleton and other organells. In fact, the mechanical properties of a cell are likely to be crucial for its migratory behavior in a given environment [5]. For example, mechanics of human pancreatic adenocarcinoma cells (Panc-1), a cellular model system expressing mainly keratin 8 and 18 as intermediate filaments, are highly dependent on the cell's keratin cytoskeleton organization. Addition of the bioactive phospholipid sphingosylphosphorylcholine (SPC) leads to a substantial modification of the keratin network towards a perinuclear rearrangement [8], [9]. This results in reduced cell stiffness and enhances the ability of cells to sequeeze through porous membranes in a Boyden chamber assay. Thus, it is suggested that SPC enhances the metastatic potential of pancreatic tumor cells [10].

**Figure 1:**
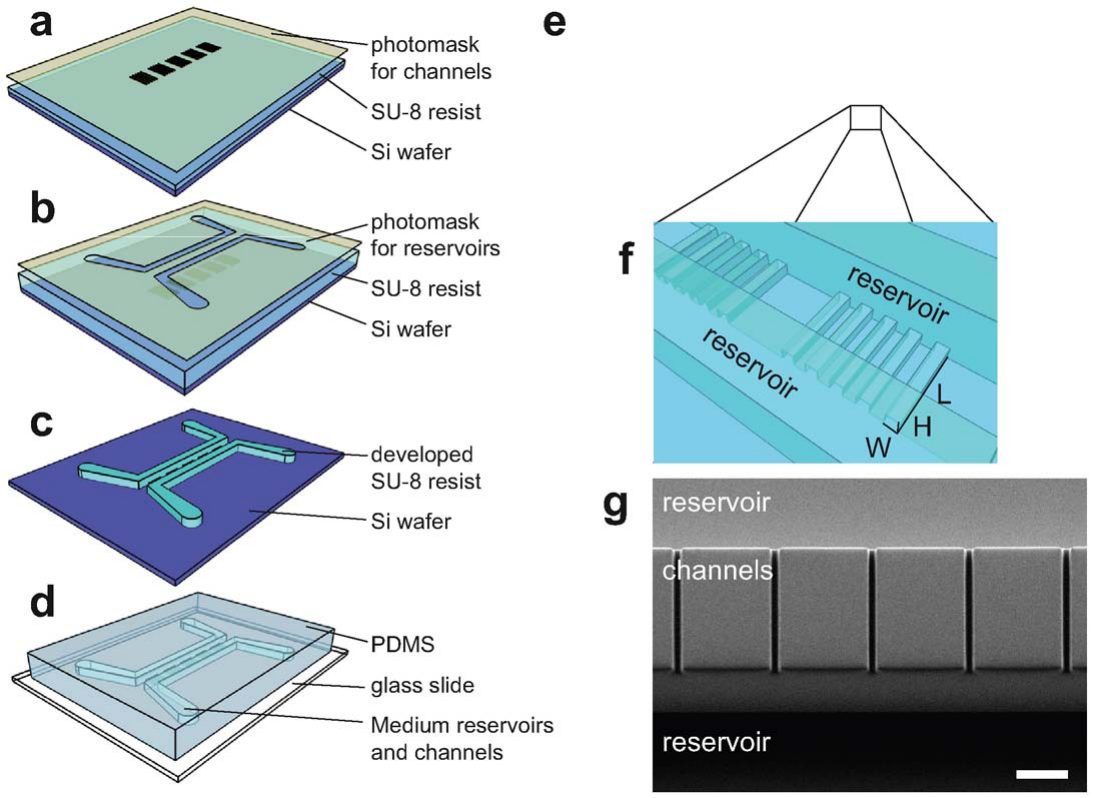
Fabrication of the migration chip. (a–d) Fabrication steps of the migration chip with a two-step photolithography process followed by a replica-molding step using (poly)dimethylsiloxane (PDMS). In the last step the cast PDMS mold is bonded to a glass cover-slide. (e) Schematic of a migration chip array for medium throughput experiments. (f) Schematic close-up of channel structures with the size-controlling parameters. (g) Scanning electron microscope image of the PDMS channels. The structures refer to channels with the size of 7×11×150 µm (WxHxL) and 150 µm high reservoirs that are connected by the cannels. Scale bar, 50 µm.

In order to investigate the invasiveness of such tumor cells into a well-defined 3D environment, we developed a migration assay whose key features are micro-sized channel structures of various widths that can be accessed by the cells. These microchannel arrays are arranged in close proximity on a glass slide in order to enable automated live-cell imaging. The fabrication of the developed migration-chip is based on simple photolithographic processes and replica-molding using a transparent elastomer as depicted in Figure 1. Using the migration chip, we analyzed cell migration inside the 3D channels and on the 2D flat surfaces in front of the channels. For Panc-1 cells treated with SPC we found an enhanced invasive migration behavior and a distinguished migration pattern in 2D and 3D. To elucidate whether the 3D aspect of the channel walls is the important factor, or only the imposed linear one-dimensional (1D) directionality of cellular movement, we performed migration experiments with microcontact printed adhesive lines.

## Results

### Micro-Fabricated Migration Chip and General Cell Behavior

**Figure 2:**
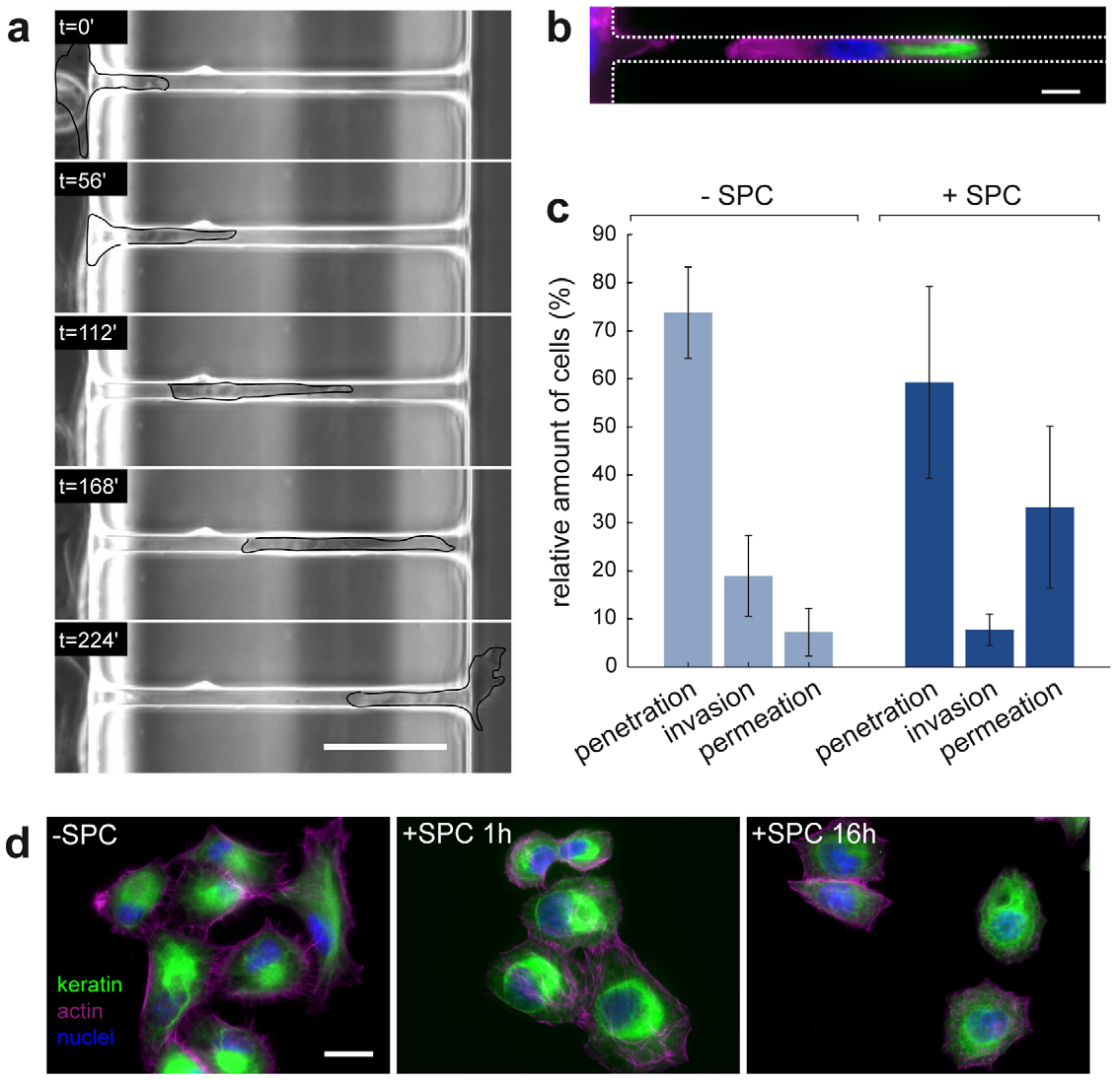
Interaction of cells with channel structures and SPC-effect on cytoskeleton structure. (a) Images of a time-lapse video with a sphingosylphosphorylcholine (SPC)-treated Panc-1 cell *permeating* a channel (7×11×150 µm). For better visualization, the cell was encircled manually. Timesteps are indicated in each image, scale bar, 50 µm. For the whole sequence see Video S2. (b) Panc-1 cell inside a channel (7×11×150 µm) migrating from left to right with labeled cytokeratin (green), actin (magenta) and nuclei (blue), non-SPC-treated. Scale bar, 10 µm. (c) Summary of cell interactions with the channels for the non-SPC-treated case (−SPC, 4 experiments, N = 100) and upon treatment with 10 µM SPC (+SPC, 4 experiments, N = 115). (d) Panc-1 cells without treatment (−SPC) and after 1 h and 16 h of incubation with 10 µM SPC. Cytokeratin (green), filamentous actin (magenta) and the nuclei (blue) are visualized by appropriate stainings. Scale bar, 50 µm.

In this work we developed a micro-fabricated device with channel structures mimicking a 3D confined environment based on soft lithography methods [11]. With this micro-fabricated device we were able to follow cell migration through precisely defined structures in real time (Fig. 1 a–d). In combination with an automatically controlled microscope we used the migration chip for screenings of migrating Panc-1 cells using transmitted light and fluorescence live-cell imaging as demonstrated exemplarily in Videos S1, S2, S3. Cells could be seeded and cultivated in the device under normal cell culture conditions. They showed the expected spreading and migration behavior on the flat surface in front of the channels where they migrated in a non-directed way. However, as soon as the cells reached the walls between the single channels, they preferred to walk along the walls, a phenomenon known as “contact guidance” [12], [13]. Once the cells approached the entrance of a microchannel, they were able to migrate through it as long as the channel cross-sections were relatively large (WxHxL: 15×11×50 µm) as shown in Video S1. This permeative migration behavior through the channels did not have a major effect on the migration speed of the cells nor were the cells forced to deform themselves in a substantial way. By reducing the channel width from 15 to 7 µm, only 7% of the Panc-1 cells that initiated contact with the channels were able to permeate them. Moreover, they had to deform themselves in a dramatic way to enter the channels (Fig. 2 a and b). At channel widths of 3 µm cells were not able to invade the channels although parts of their cytoplasm extended into it (supplementary Figure S1). In order to estimate the volume that a cell occupies inside the channel, we calculated the corresponding cell volume from the diameter of cells in suspension (19±2 µm). For the channels with a width of 7 µm this means that an average cell that moves completely inside the channel will occupy one third of the channel volume which leads to a 1.6 fold increase in cells surface area due to the changed geometry from a sphere to a rectangle, assuming that the cell volume remains constant.

### SPC Alters Spatial Keratin Organization, 2D Cell Migration, and Invasive Cell Behavior

Treatment of Panc-1 cells with 10 µM SPC leads to a perinuclear organization of the cellular keratin cytoskeleton (Fig. 2 d) and enhances their motility. We measured the migration speed on the flat area inside the migration chip of non-treated (0.32±0.04 µm min^−1^; N = 195) and SPC-treated (0.47±0.1 µm min^−1^; N = 252) Panc-1 cells. The migration speed showed a significant 1.5-fold increase upon treatment of cells with SPC.

In order to describe the behavior of the cells that initiated contact with the channels, we assigned them to one of the following three categories: (i) Cells that penetrated the channels with their cytoplasm to a depth of at least 20 µm were classified as *penetrating* cells. (ii) Cells that completely entered the channel structure and then stopped migrating or turned around were called *invasive* cells. (iii) Cells that migrated completely to the other side of the channel were termed *permeative* cells (see also Materials and Methods section for more details). An example of a cell *permeating* a microchannel is shown in Figure 2 a (examples of all three different behaviors can be seen in Video S2). The reorganization of the cell's keratin and actin network as well as the deformed nucleus inside the channels can be seen in Figure 2 b. The categorized behavior of cells that interacted with the microchannels is summarized in Figure 2 c. In the absence of SPC, the amount of cells that *invaded* or *permeated* the channels was relatively low (19% and 7%, respectively) as compared to the 73% of cells that only *penetrated* the channels while their nuclear region stayed outside the channels. This ratio changed significantly once the cells were treated with 10 µM SPC. The addition of SPC resulted in a highly increased *permeative* behavior of the cells. 33% of the cells permeated the channels in response to SPC-treatment, a 4.7-fold increase over the non-treated cells. These differences in cell behavior are remarkable, given that not 100% of the cells respond to SPC treatment as determined by examining optically the keratin organization of immunostained cells. In the absence of SPC, about 33% of the Panc-1 cells exhibited a predominantly perinuclear organization of keratin 8/18 filaments. This number increased to about 66% in the presence of the bioactive lipid in accordance with previous data [9]. To elucidate if the nucleus size played a role in determining the cell's invasive behavior, we measured the nucleus diameters of cells, with respect to SPC treatment, on a flat surface. The non SPC-treated cells had a nucleus diameter of 17.0±0.4 µm (N = 77), and cells exposed to SPC for 1 h and 4 h had nucleus diameters of 14.9±0.17 µm (N = 196) and 15.9±0.27 µm (N = 75), respectively. Thus, the nucleus diameter is reduced by 12% after one hour of SPC-treatment but at 17.0 µm it is still too big to fit into channel with a cross-section of 7×11 µm without deforming itself.

### 3D Channel Structures Have an Impact on Migration Dynamics

**Figure 3:**
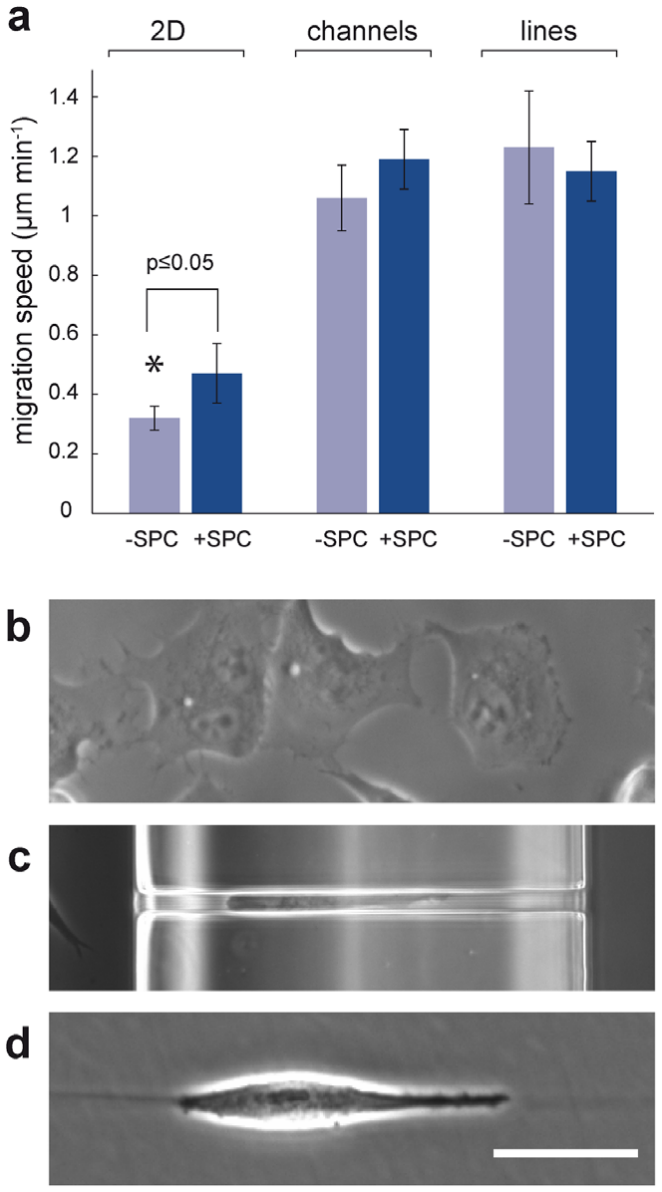
Migration speeds in different environments. (a) Comparision of the migration speed of cells migrating either on a flat 2D glass surface, through microchannels or along adhesion mediating lines with respect to the presence of SPC. The average speed of Panc-1 cells on a flat glass surface (2D) is increased upon treatment with 10 µM SPC. Migration speed in microchannels is generally higher but not affected by SPC-treatment as well as the one along lines; error bars show s.e.m. (b–d) Examples of cells migrating on a flat surface (b), inside a channel (c), and along a adhesion mediating lines (d). Scale bar, 50 µm.

We analyzed and compared cell migration on flat surfaces (2D) with migration inside channels (3D). In order to elucidate whether the 3D architecture of the channel walls or only the restriction to 1D motion changed the migration phenotype of the cells, we additionally observed the Panc-1 migration behavior along adhesive lines printed on a non-adhesive surface (Video S4). Comparing the non-treated 2D migration speed (0.32±0.04 µm min^−1^) with that inside the channels, we found an approximately three-fold increase in migration speed inside the channels for both, non-SPC-treated (1.06±0.1 µm min^−1^; N = 13) and SPC-treated cells (1.19±0.1 µm min^−1^; N = 26). Thus, migration speed inside the channels was not significantly increased upon SPC-treatment. Cell migration speed along the lines was slightly higher than inside the channels (1.2±0.19 µm min^−1^; N = 12) and also not significantly influenced by SPC treatment (1.15±0.01 µm min^−1^; N = 13). The different migration speeds are summarized in Figure 3 a–d.

**Figure 4:**
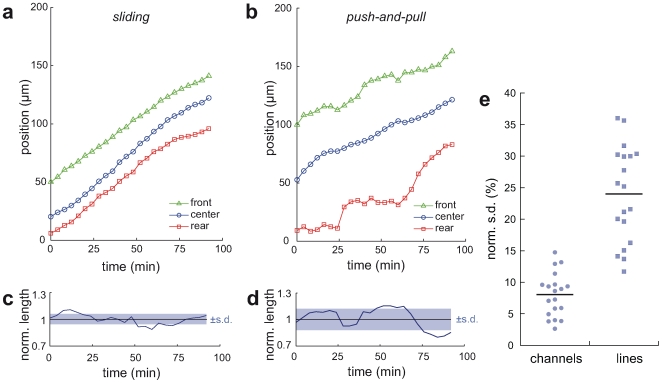
Cell migration dynamics inside channels and on lines. (a) Typical profile of a *sliding*-like migration pattern inside a channel where the cell's front, center, and rear are moving in a synchronous, almost equidistant manner (b) Typical profile of a *push-and-pull*-like migration behavior on adhesion mediating lines where the cell's front and rear position changes in an oscillating manner independent of the nucleus displacement. (c, d) Length variation of cells during migration, normalized to the mean cell length (black line). Standard deviations (s.d.) of the mean length are indicated by the shaded rectangle. *Sliding*-like migration is charcterized by smaller s.d.s (c) than the ones obtained for *push-and-pull*-like patterns (d). (e) Distribution of normalized s.d.s of single cells performing a directed migation inside channels or along lines.

To investigate whether distinct compartments of the tumor cells show coordinated movement, we separately tracked the leading edge, nucleus, and rear edge of *permeating* cells. Plotting the absolute position inside the channel versus time, two characteristic patterns of motion can be distinguished: a smooth *sliding* motion and a stepwise *push-and-pull* behavior (Fig. 4 a and b, respectively). The *sliding* pattern is characterized by an equidistant movement of the front and rear of the cell. This was quantified by calculating the normalized standard deviation (s.d.) from the mean cell length. An example of variation in cell length normalized to its mean length is given in Figure 4 c. For this *sliding* behavior of cells inside channels this normalized s.d. was found to be 8% (N = 19). In contrast, the *push-and-pull* migration pattern is characterized by a variation of the cell length in an oscillating manner (Fig. 4 d), similar to the classical stepwise migration pattern of fibroblasts on a flat surface [14]. This leads to an increased normalized s.d. of the mean cell length with a value of 24% (N = 19). Figure 4 e summarizes the obtained data demonstrating the two distinct migration patterns.

## Discussion

With our migration chip, channel structures of sub-cellular dimensions have been applied to study cell deformation and invasiveness as well as migration dynamics and coordination of cellular movement. In addition, our system is well suited for fluorescence live-cell imaging. Due to its precise channel architecture, it allows for a quantitative analysis of mechanical deformation of cell migration through confined environments. In the last few years, similar channel architectures for studying leukocyte migration inside a confined 3D environment have been used [15], [16]. Very recently Irimia *et al*. [17] examined the migrational persistence of cancer cells inside micro-sized channels, demonstrating the usefulness of such single-cell based approaches.

Using the functionality of our migration chip, we address the question whether the known effect of SPC on Panc-1 cells (decreasing the cells' stiffness) also enables the cell-driven invasion into confined spaces. Beil *et al*. measured a drop in the Panc-1 cells' elastic modulus from about 28 to 16 mN m^−1^ upon SPC-treatment using a micro-plate based single-cell stretcher [9]. Using a Boyden chamber assay they correlated this decrease in stiffness to an increased ability of the cells to squeeze through the membranous pores. We characterized and quantified the behavior of cells initiating contact with channels of a cross-section of 7×11µm, a size at which the Panc-1cells were hardly able to squeeze through. With these channel architectures we were not only able to confirm the result of Beil *et al*. as we observed a five-fold increase in the number of cells permeating the channels upon SPC-treatment but could additionally observe and quantify the migration dynamics inside the channels.

The enhanced invasive behavior upon treatment with SPC may be explained by using a simple two-component model. First, there is the motor unit with the driving actin-polymerization (lamellipod) in the front and the contractive acto-myosin assembly at the rear of the cell. Secondly, there is the passive and voluminous cell body being pulled upon migration. For an *invasion* into the channel, the cell needs to deform the cell body requiring compression of the keratin envelope and the nuclear region. Inside the channels, nucleus shape and keratin network structure deviate from the normal spatial distribution in the cell as shown in Figure 2 b. Assuming that Panc-1 cells are able to generate only a finite force, we speculate that the motor unit of a non-SPC-treated Panc-1 cell is not able to pull the cell body into a channel with a width of 7 µm. This speculation is supported by observations on neutrophil leukocytes migrating inside small capillaries. These cells are able to counterbalance a maximal hydrostatic counter-pressure of 1.5 kPa by generating a traction force of 38 nN inside the capillaries [18]. Additionally, it was shown for leukocytes that the spatial organization of intermediate filaments plays a major role for the cells' migratory behavior [19].

Beyond the effects of size exclusion on the invasive behavior of cells, our migration chip permitted the quantification of drastic differences in 2D and 3D migration speeds. This property is of particular importance since recent studies attributed increases in migration speed to the dimensionality of the cell environment [1], [20]. Our observed increase in 2D speed after SPC-treatment might be attributed either to the keratin reorganization and softening of the cell or to the known enhancement of filamentous actin formation by SPC [8]. As the speed inside the channels is not affected by SPC-treatment we speculate that the SPC-effect on actin may not be the major factor, but that the different keratin morphology might cause the increased migration speed on flat surfaces. Assuming that the nuclear region and keratin envelope of a cell inside a channel is in a compacted state, its steric hindrance should have only a minor influence on the migration, independent of SPC-treatment. If SPC-enhanced actin dynamics were the predominant reason for the observed increase in migration speed on 2D surfaces, this should also lead to an increased speed inside the channels which we did not observe. Thus, SPC facilitates the initial invasion into the channel but seems not to affect the further migration speed inside the 3D environment.

Recent research has identified different migration phenotypes in 2D and 3D [1], [2], [20]. In particular, an adhesion independent migration mechanism has been reported for leukocytes in a 3D matrix [2]. Based on a theoretical model such a migration mechanism may be attributed only to partial pressure differences between the leading and rear edge without the necessity of specific cell-surface adhesion [21]. With our migration chip we did not test this particular migration mechanism but we could reveal two distinct migration phenotypes that are proposed to occur in either a 2D or 3D environment. Doyle *et al*. observed a coordinated migration in fibroblasts moving on thin (1D) lines with a width of up to 5 µm [1]. They argue that this behavior depends exclusively on the width of the adhesive lines and resembles the behavior in 3D. Similarly, we found in our experiments that the majority of the cells showed a 2D-typical *push-and-pull*-like movement when migrating on 7 µm wide lines. However, inside the channels with the same width the majority of the cells showed a *sliding*-like movement. Therefore, we propose that the transition from a 2D to a 3D or 1D migration mechanism depends not only on the line width but is also determined by the contact area with the environment, in our case the channel walls.

In summary, we report a detailed investigation of migration dynamics of human pancreatic cancer cells inside micro-channels with a particular focus on the effects of keratin reorganization induced by the bioactive phospholipid SPC. Beyond previous knowledge, we demonstrate that the SPC treatment of Panc-1 cells increased their ability to *invade* and *permeate* narrow channels. Hence, our study may contribute to a more detailed understanding of how cancer cells invade the surrounding tissue and also escape from a primary tumor (*permeate* through the stroma), the first step in tumor spreading. Furthermore, we demonstrated the existence of two different migration phenotypes depending on the dimensionality of the cell environments. Thus, our migration chip provides an easy to use experimental to promote current research on 3D migration behavior on a single-cell level.

## Materials and Methods

### Fabrication of Migration Channels

A two-step photolithography method was used to fabricate the migration microchannels as sketched in Figure 1 a–d. Parameters for the photolithography process were similar to those reported by Greiner *et al*. [22]. The first, thinner layer of SU-8 10 photoresist (MicroChem, Newton, MA) was spin-coated defining the height of the channels (11 µm). After soft baking, exposure with the first chrome mask which defined the channel widths (7–15 µm), and the post exposure bake, a second, much thicker, layer (150 µm) of SU-8 2075 photoresist (MicroChem, Newton, MA) was spin-coated on the first layer. Exposure with a second mask allowed the building of two reservoirs for media on both sides of the channels and thereby also determined the channel length (150 or 50 µm). The reservoirs were relatively high (150 µm) in order to provide the cells with enough media for long-time experiments (16 h) without any flow applied. The chrome masks used for the exposure were custom made and obtained from Masken Lithographie & Consulting (Jena, Germany). After the post-exposure bake the structured wafer was developed and hard baked. The structured wafer served as a master for the subsequent replica molding process with (poly)dimethylsiloxane (PDMS; Dow Corning, Midland, MI). The PDMS pre-polymer was mixed with the cross-linker at a ratio of 10∶1, cast on the master, and polymerized for 4 h at 65°C. The polymerized PDMS mold was then covalently bound on a glass cover slide after oxygen plasma activation of the surfaces (0.1 mbar O_2_ using 150 W for 20 s). Channel geometry and dimensions of the cast PDMS were verified using scanning electron microscopy (Fig. 1 g) and conventional optical microscopy. The size of a single migration chip assembled on a cover slide was 2×3 cm.

### Microcontact Printing of Fibronectin Lines

For the study of cell migration on lined structures, ultra-low attachment culture dishes (Corning, Lowell, MA) were decorated with fibronectin lines. The lines were microcontact printed using a PDMS stamp. A 50 µg ml^−1^ fibronectin solution (Invitrogen, Karlsruhe, Germany) was used for the printing with 20% of the fibronectin labeled with Atto488 (ATTO-TEC, Siegen, Germany) in order to visualize and control the printing efficiency.

### Cell Culture

All chemicals, unless otherwise noted, were purchased from Invitrogen (Karlsruhe, Germany). Human pancreatic epithelial cancer cells (Panc-1) (European Collection of Cell Cultures ECACC) were cultured in Dulbecco's modified Eagle's medium containing 4.5 mg ml^−1^ glucose and supplemented with 10% (vol/vol) fetal bovine serum (FBS) (PAA Laboratories, Cölbe, Germany), 1% (vol/vol) L-glutamine, and 1% (vol/vol) Penicillin-Streptavidin. Cells were cultivated in 75 cm^2^ culture flasks at 37°C and 5% CO_2_. Cells were subcultured by treating them for 3–4 min with 0.25% (vol/vol) trypsin-EDTA. Cell numbers and diameters were counted and measured by the Coulter Counter Z2 (Beckmann Coulter, Krefeld, Germany). T. Busch (University of Ulm) provided us with stably transfected Panc-1 cells labeled with keratin K8-eCFP/K18-eYFP.

### Immunostaining

For cell stainings, the cells were plated on fibronectin-coated glass cover-slips and fixated by incubation with a 3.8% (wt/vol) para-formaldehyde solution for 10 min at 37°C. After washing with phosphate buffered saline (PBS), they were treated with 0.5% (vol/vol) TritonX-100 (Carl Roth, Karlsruhe, Germany) in PBS for 10 min at room temperature and washed again before incubation with 0.2% (vol/vol) gelatin from fish skin in PBS for 10 min at room temperature. For the immuno-staining of the keratin network, the Cytokeratin Pan antibody (Imgenex, San Diego, CA) was diluted (1∶200) in a 0.5% TritonX-100/0.2% gelatin from fish skin solution. After rinsing with PBS, the cells were stained with the following solution for 2h at room temperature: Alexa Fluor 488 chicken anti-mouse (1∶1000), Alexa Flour 568 phalloidin (1∶50), and 0.2 µg ml^−1^ Hoechst 33342 dissolved in 0.5% TritonX-100/0.2% gelatin from fish skin. The stained cells were glued with Aqua-Poly/Mount Medium (Polysciences, Eppelheim, Germany) to a cover slide.

### Migration Experiments

The channel system of the migration chip was gently flushed with a solution of 70% (vol/vol) Ethanol and deionized water for sterilization followed by deionized water and, finally, phosphate buffered saline (PBS). Surfaces inside the chip were coated with fibronectin. A 50 µg ml^−1^ fibronectin solution was injected into the chip and incubated overnight at 4°C for physisorption. Before cells were seeded, the fibronectin solution was exchanged for cell culture medium and heated for 30 minutes in the incubation chamber of the microscope at 37°C in order to adjust to the higher temperature. Prior to the migration experiments, cells were detached from the surface by treating them with 0.25% (vol/vol) trypsin-EDTA. After centrifugation, the cell pellet was resuspended with medium containing 5% (vol/vol) FBS with a final concentration of 1–2×10^6^ cells ml^−1^. Approximately 100 µl of the cell suspension were gently introduced into the chip to seed the cells in close proximity to the channels. SPC, with a final concentration of 10 µM, was added to the medium once the cells adhered to the surface; image capturing for time-lapse videos was started 60 min after addition of SPC. Phase-contrast live-cell imaging was performed in a heated and air-humidified chamber built around an automated inverted microscope (Axiovert 200M; Carl Zeiss, Jena, Germany) and controlled with the AxioVision software (AxioVision V 4.6; Carl Zeiss, Jena, Germany). The motorized stage enabled the observation of 40 channels in one experiment. For time-lapse videos, images were taken every four minutes at each position over a period of 16 h.

### Image and Data Analysis

Images were processed and analyzed using the ImageJ software (http://rsbweb.nih.gov/ij/). The manual tracking plug-in was used to follow the migration of the cells inside the channels on flat surfaces or on adhesion mediating lines. The data obtained from the cell tracking were processed using routines written with MATLAB (Version 7.5, The MathWorks, Natick, MA). To determine the nuclei diameters, cells were stained with 0.2 µg ml^−1^ Hoechst 33342 and from the fluorescent area an average diameter was calculated.

In order to completely characterize the different behaviors of the cells that contacted the channels, each instance was assigned to one of the following three different categories. (i) Cells that penetrated the channels with their cytoplasm to a depth of at least 20 µm were classified as *penetrating* cells. This minimum limit of penetration depth was chosen in order to exclude cells that were moving perpendicular to the channel direction without a change in their directionality (8% of the total number of interacting cells for the -SPC and 12% for the +SPC condition). (ii) All the cells that completely entered the channel structure and then stopped migrating or turned around were called *invasive* cells. (iii) Cells that migrated completely to the other side of the channel were termed *permeative* cells. Finally, cells that were still migrating through the channels when the image acquisition stopped after 16 hours (13% for -SPC and 27% of the cells for +SPC) were excluded from classification, as it was not possible to determine their assignment between categories (ii) and (iii).

The cells' mean migration speeds were measured by tracking the displacement of the moving cell from frame to frame in a time-lapse experiment. The cell's mean speed was then calculated from the obtained Euclidean distances over a time period of at least 3h. Analysis of the dynamical change in cell length of cells migrating inside the channels and on adhesive lines was carried out by plotting the cell length over time and calculating its mean value within comparable time frames. We set the mean cell length to one and plotted the respective normalized standard deviations (s.d.). Low values of the normalized s.d. refer to low fluctuations in the cell length (smooth *sliding*) while higher values represent higher fluctuations (*push-and-pull* like pattern).

Statistical analysis was carried out using MATLAB (Version 7.5, The MathWorks, Natick, MA) and Kaleidagraph (Version 4.0, Synergy Software, Reading, PA). Errors are given as standard error of means (s.e.m.) if not differently indicated.

## Supporting Information

Figure S1 Panc-1 cell with fluorescently labeled keratin 8 and 18 penetrating a channel (3×11×150 µm). The cell membrane inside the narrow channel is highly deformed while the spherical nucleus is located outside at the channel's entrance.
(0.59 MB TIF)

Video S1 Panc-1 cells seeded in front of channels with a width of 15 µm. Cells migrate on the flat surface in front of the channels and are able to permeate the channels without major deformation. Channel dimensions are 15×11×150 µm (WxHxL), time-lapse video of 16 hours played in 3000× real-time. Scale bar, 50 µm.
(1.81 MB MOV)

Video S2 Representative examples of Panc-1 cells in front of the channels that show penetrative, invasive, and permeative behavior. Channel dimensions are 7×11×150 µm (WxHxL), time-lapse sequences of 6:34 h played in 3000× real-time. Scale bar, 50 µm.
(0.67 MB MOV)

Video S3 Live-cell video of Panc-1 cells with fluorescently labeled keratin 8 and 18 migrating through a channel. Channel dimensions are 15×11×150 µm (WxHxL), time-lapse sequences of 16:00 h played in 4300× real-time. Scale bar, 50 µm.
(0.55 MB MOV)

Video S4 Panc-1 cells migrating along microcontact printed lines of fibronectin on a non-adhesive surface. Line width is 7 µm, time-lapse video of 6:34 h played in 3000× real-time. Scale bar, 50 µm.
(0.43 MB MOV)
