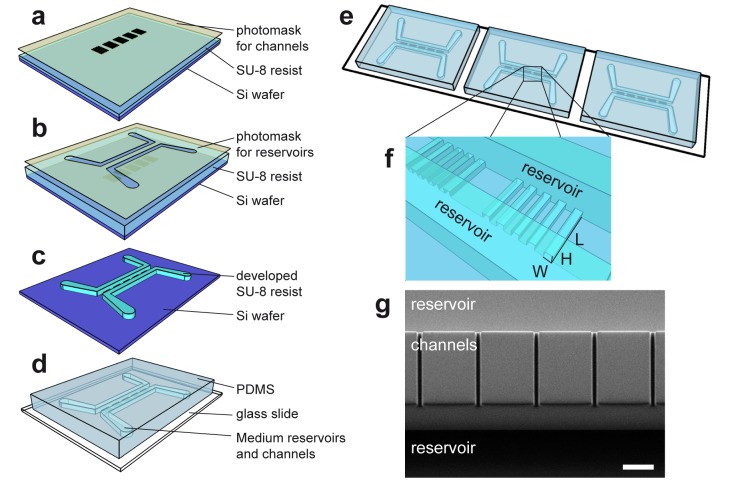# Correction: Impact of Tumor Cell Cytoskeleton Organization on Invasiveness and Migration: A Microchannel-Based Approach

**DOI:** 10.1371/annotation/9a6b2508-81c8-403f-87bd-071bdcb5b251

**Published:** 2010-03-01

**Authors:** Claudio G. Rolli, Thomas Seufferlein, Ralf Kemkemer, Joachim P. Spatz

There was an error in Figure 1. Please view the corrected Figure 1 at: 

**Figure:**