# Supplementary figures and images for: Impact of Tumor Cell Cytoskeleton Organization on Invasiveness and Migration: A Microchannel-Based Approach
Source: PLoS One. 2010 Jan 15;5(1):e8726. doi: 10.1371/journal.pone.0008726 (PMC2806915; doi:10.1371/journal.pone.0008726)

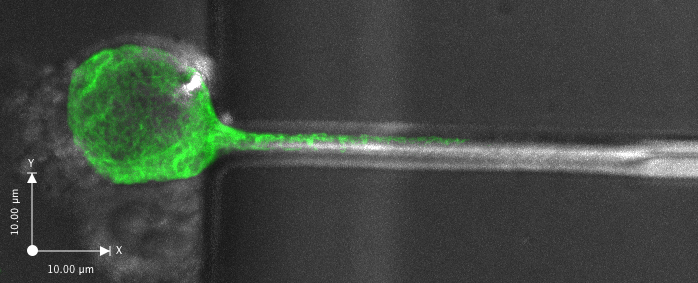

Supplement: Figure S1 — Panc-1 cell with fluorescently labeled keratin 8 and 18 penetrating a channel (3×11×150 µm). The cell membrane inside the narrow channel is highly deformed while the spherical nucleus is located outside at the channel's entrance. (0.59 MB TIF) [file pone.0008726.s001.tif]
